# Umami as an ‘Alimentary’ Taste. A New Perspective on Taste Classification

**DOI:** 10.3390/nu11010182

**Published:** 2019-01-16

**Authors:** Isabella E Hartley, Djin Gie Liem, Russell Keast

**Affiliations:** Centre for Advanced Sensory Science, School of Exercise and Nutrition Sciences, Deakin University, 1 Gheringhap Street, Geelong 3220, Australia; iehartle@deakin.edu.au (I.E.H.); gie.liem@deakin.edu.au (D.G.L.)

**Keywords:** basic tastes, taste, taste reception, umami

## Abstract

Applied taste research is increasingly focusing on the relationship with diet and health, and understanding the role the sense of taste plays in encouraging or discouraging consumption. The concept of basic tastes dates as far back 3000 years, where perception dominated classification with sweet, sour, salty, and bitter consistently featuring on basic taste lists throughout history. Advances in molecular biology and the recent discovery of taste receptors and ligands has increased the basic taste list to include umami and fat taste. There is potential for a plethora of other new basic tastes pending the discovery of taste receptors and ligands. Due to the possibility for an ever-growing list of basic tastes it is pertinent to critically evaluate whether new tastes, including umami, are suitably positioned with the four classic basic tastes (sweet, sour, salty, and bitter). The review critically examines the evidence that umami, and by inference other new tastes, fulfils the criteria for a basic taste, and proposes a subclass named ‘alimentary’ for tastes not meeting basic criteria.

## 1. Introduction

The relationship between individual variation in taste perception, food choice and intake, and ultimately diet related disease, provides a framework for applied taste research. A taste perception arises from the interaction of non-volatile, saliva soluble chemicals with taste receptors on the tongue within the oral cavity. This interaction initiates a signal transduction to processing regions of the brain, resulting in the formation of a taste perception. The taste perception formed could include the perception of one of the basic tastes: sweet, sour, salty, bitter, umami [1], or fat [2]; or a perception of other putative taste qualities including, but not limited to, kokumi (rich, mouthful, thick, delicious taste) [3,4], carbohydrate [5], calcium [6], or metallic tastes [7]. Detection and perception of basic tastes is hypothesised to exist for species’ survival throughout evolution to prevent the consumption of potential noxious food, and promote consumption of nutritious food, a nutrient–toxin detection system [8,9,10,11]. 

Basic tastes have perceptual independence, that is, they do not elicit a taste perception similar to that of any other basic tastes and cannot be produced from a combination of other tastes [12,13,14]. The concept of basic tastes dates back as far 384–322 B.C. when Aristotle originally listed the seven tastes he proposed as basics, these included sweet, sour, salty, and bitter as well as astringent, pungent, and harsh [15]. Throughout history the lists of tastes have been extended, or reduced, depending on the prevailing thought of the time, with the only consistency being the inclusion of sweet, sour, salty, and bitter in basic taste lists [15]. Research during the 1800’s separated olfaction and tactile perceptions from tastes. The development and advancement of science and technology, including psychophysical testing led to sweet, sour, salty, and bitter tastes being confirmed as basics as evidenced throughout published literature [16].

Advances in molecular biology, and the recent discovery of taste receptors and ligands has increased the basic taste list to include umami and fat taste [17,18,19,20]. The existence of specific taste receptors responsive to a single compound that elicits a taste is often suggested as a key piece of evidence for basic tastes classification, with literature citing this as the key evidence in the case of umami [14]. Due to these recent advances, there is the potential for a plethora of new tastes to be discovered, and potentially classified as a basic taste if receptors on taste cells are found to respond to ligands. The current possibilities include, but are certainly not limited to kokumi, carbohydrate, calcium, and metallic tastes.

Thus, due to advances in knowledge, and the possibility for an ever-growing list of basic tastes, it is pertinent to critically evaluate whether new tastes, including umami, are suitably positioned with the four classic basic tastes (sweet, sour, salty, and bitter). This is of importance for three predominant reasons, first, there is historical and academic relevance to determine whether umami, and by inference other new tastes, should in-fact be considered in the same category as sweet, sour, salty, and bitter. Second, understanding individual variation in taste perception, perceptual associations with other basic tastes, and physiological responses resulting from detection of specific tastants, may enhance our understanding of the complex relationship between taste, dietary choice and intake, and diet related health related outcomes. Third, the classic basic tastes have significant immediate influence on whether to swallow or not swallow a potential food, while the more recent tastes such as umami and fat may have more post-ingestive relevance and determine extent of consumption and ultimately health. The review that follows will critically evaluate the evidence that umami fulfils criteria for classification as a basic taste.

## 2. Basic Taste Criteria

Criteria that a stimulus must fulfil for it to be classified as a basic taste has been proposed, although these criteria have not been consistent [12,13]. Kurihara and Kashiwayanagi (2000) suggested that for a compound to be considered a basic taste it should fulfil the following criteria. The proposed basic taste is (1) different to any other basic taste; (2) not replicated by combining other basic tastes and; (3) a taste which is commonly consumed and induced by common components of food [13]. The requirement for a basic taste to have an identified receptor was recently added to this set of criteria [14]. The argument could then be put forward that the discovery of a receptor-ligand complex alone is not reason to justify a taste as a basic taste. Whether the detection of the stimulus from that receptor is transduced and forms a unique perceptible experience may be of higher importance. That is, if a taste receptor is identified, but no unique perceptible experience occurs from the activation of that receptor, then is it appropriate to classify the stimulus as eliciting a basic taste? 

A more comprehensive set of criteria have been outlined, covering both unique effective stimuli, transduction (receptors), neurotransmission, and finally, perception [12]. These criteria have been used previously to investigate the appropriateness of other new tastes, specifically fat taste; in this review the following criteria will be used to specifically investigate umami as a basic taste [10,12]. We extend this criterion to involve hedonic responses occurring from tasting the effective stimuli. The criteria are as follows:

1. A distinct class of effective stimuli must exist.
2. Detection of effective stimuli must have an evolutionary benefit.
3. Transduction mechanisms that can convert the chemical code of the effective stimuli into an electrical signal, including receptors, must exist.
4. Neurotransmission of this electrical signal to taste processing regions of the brain must occur.
5. Perceptual quality arising from this processing must be independent from other taste qualities.
6. Hedonic responses occur from taste perception.
7. Physiological and/or behavioural responses must occur following the activation of taste bud cells by the effective stimuli.

## 3. Umami Taste and Unique Class of Umami Effective Stimuli

Umami was initially discovered by Ikeda who isolated glutamic acid from kombu (seaweed), finding that the salts of glutamic acid, particularly the sodium salt, monosodium glutamate (MSG), gave the seaweed its specific flavour (translated in [21]). Thus, free l-glutamate (glutamic acid) is the predominant umami effective stimuli, and MSG is the predominant prototypical umami stimuli used in psychophysical testing. It was later discovered that the taste of l-glutamate could be synergistically increased through the addition of disodium 5’ribonucleic acids, specifically disodium 5’inosinate monophosphate (IMP) and disodium 5’guanylate monophosphate (GMP) [22]. When tasted in isolation IMP elicits a minimal to weak umami taste hypothesised to occur due to the interaction of IMP with subthreshold concentrations of l-glutamate in humans’ saliva, demonstrating that IMP and GMP require l-glutamate for an umami taste perception to occur [23]. In human psychophysical studies, the addition of 0.5 mM IMP significantly reduces the concentration of MSG required for participants to reach RT (7.66 mM and 0.20 mM MSG respectively) due to the taste potentiation produced when IMP is applied with MSG [24]. 

Free l-glutamate is naturally present in high concentrations in a wide variety of foods including certain vegetables (and fruits i.e., tomato), seaweeds, aged cheese, seafood, fish and soy sauce, egg yolks, and human breast milk [14,25,26,27]. Whereas, IMP is found predominately in animal products such as chicken, pork, beef, and tuna, and GMP in dried mushrooms such as shitake [14,25,26]. The curing, ageing, heat treatment, and fermenting of certain foods results in an increase in free amino acids, including l-glutamate, and often an increase in umami potentiating ribonucleotides (IMP and/or GMP) [14,26]. Specifically, in animal products such as beef, pork, chicken, and fish that contain high concentrations of protein, which is essentially tasteless, proteolysis occurring from fermentation, curing, or heat treatment releases a complex mixture of amino acids, including l-glutamate. As these animal products naturally contain high concentrations of IMP the umami taste potentiation between IMP and l-glutamate can occur [14,26]. For example, during the process of ageing beef the concentration of free l-glutamate has been shown to increase by approximately 33% over eight days, and when this is combined with naturally present IMP, umami taste potentiation can occur [26]. 

The increase of tastants occurring from fermenting, curing, heat treatment or ageing is not unique to specifically umami taste quality, with kokumi taste (rich, mouthful, thick, delicious taste) peptides similarly increasing through these same processes. Kokumi tasting compounds include certain γ-glutamyl peptides and during the ageing of dairy products [28] and in fermented products including soy or fish sauce [4,29] the concentrations of γ-glutamyl peptides increases. This ageing/fermenting process results in an accumulation of peptides including γ-glutamic acid that forms a peptide bond with an amino group of a non-polar amino acid [30]. Additionally, glutathione (GSH) a tripeptide made up of glutamic acid, cysteine, and glycine, which elicits a kokumi taste, similarly increases in concentration through fermentation of certain foods. Kokumi stimuli including GSH, similar to IMP, elicit little taste in isolation, but when combined with an umami solution enhances the mouthful, continuity, and thickness, thus enhancing the kokumi aspects of the umami solution [4]. Similar to umami effective stimuli, GSH and other γ-glutamyl peptides are common in a number of high protein foods, such as beef, chicken, and ham, and in low protein foods such as tomato juice, and red wine, at concentrations above GSH DTs [4]. This suggests that there is an association between the effective stimuli eliciting both umami and kokumi tastes in food, namely the involvement of glutamic acid derivatives in both umami and kokumi effective stimuli [30]. 

## 4. Umami Taste from an Evolutionary Perspective

In humans, the ability to detect chemicals in the oral cavity prior to ingestion, and interpret salient perceptions of sweet, sour, salty, and bitter allows for rapid evaluation of a food, identifying whether it is acceptable (swallow), or unacceptable and potentially harmful (expectorate), which was essential for species survival [11,31,32,33]. Sweet taste is stimulated by simple carbohydrates, believed to indicate the presence of readily usable energy, eliciting appetitive hedonic responses and thereby encouraging consumption [11,31]. Similarly, the detection of complex carbohydrates may also encourage consumption by signaling information regarding the carbohydrate and energy content of food [20]. Excess bitterness indicates the presence of potential toxins within food, ultimately encouraging rejection of the food [8,11,31]. Excess sourness can indicate off or spoilt foods, and is avoided to ensure the body’s acid–base balance is maintained [8,31]. Salt taste perception is posited to be for maintenance of the body’s electrolyte balance [9], for example, at high concentrations salt taste may play a role in the immediate analysis of whether to swallow or expectorate food, perhaps to avoid acute disturbance in the body’s osmotic balance [11]. The ability to detect fat taste may be less important for the rapid evaluation of food and more closely related to activating physiological responses related to digestion and food intake regulation [18,34]. 

Umami taste has previously been hypothesised to signal the presence of amino acids and protein, promoting consumption of certain protein containing foods. Conversely, many foods naturally high in free l-glutamate are not typically high in protein, for example, peas, corn, red grapes, and tomatoes [14,35]. Along the same line, high protein foods, including beef, pork, and chicken do not contain high concentrations of free l-glutamate [26]. As previously mentioned, protein is essentially tasteless, it is the proteolysis of protein within these foods occurring from fermentation, curing, or heat treatment, that releases amino acids and peptides that can stimulate taste responses. Thus, umami taste perception may indicate the presence of accessible, rather than protein bound amino acids, in foods that have been released during proteolysis occurring through various cooking processes [11]. During proteolysis it is important to consider that the amino acids released are not solely umami tasting (l-glutamate), the release of bitter tasting (i.e., L-Leucine, -Phenylalanine, -Tryptophan) and sweet tasting (i.e., L-Glycine, -Alanine, -Proline) amino acids also occurs in different concentrations depending on the specific food [36]. Thus raising the question of whether it is appropriate to designate the evolutionary purpose of umami taste perception to signal protein content of food, when proteolysis in certain foods results in a complex mixture of taste active amino acids, including sweet and bitter tasting amino acids. 

Following on from this, nutritional status, specifically protein-calorie deficiency does not appear to feedback onto preferences for umami tasting stimuli, as both malnourished (protein-calorie malnourished) and healthy infants showed preference for soup containing MSG to the same soup without MSG [37,38]. When the soup was provided in combination with casein hydrolysate, which contains a mixture of amino acids where a bitter taste dominates, the malnourished infants preferred the casein hydrolysate soup whereas the healthy infants did not [38]. Protein deficiency in infants increases consumption of protein containing food independent of the taste profile; the hypothesis that umami taste exists to signal the presence of protein is not supported. 

Glutamate receptors (T1R1/T1R3 and mGluR1) exist throughout the gastrointestinal tract [39,40], and stimulation of these receptors has been suggested to affect nutrient absorption through regulating satiety hormones including cholecystokinin (CCK) [40,41,42]. Moreover, consumption of umami stimuli (MSG) appears to be involved in appetite stimulation and satiety regulation regardless of the macronutrient (i.e., protein and carbohydrate) consumed in human behavioural studies [43] (for further discussion please see section *Behavioural and physiological responses to umami effective stimuli*). Perhaps umami is less involved in the rapid analysis of food in the oral cavity, and more involved in increasing appetite to promote consumption, whilst simultaneously assisting in regulation of protein digestion through signaling mechanisms that promote gastric secretion.

## 5. Unique Receptor and Neural Transmission of Umami Effective Stimuli

### 5.1. Unique Receptors for l-glutamate

As previously discussed, taste receptors on the tongue detect saliva soluble, non-volatile chemicals from foods in the oral cavity. Of the basic tastes, sweet, bitter, and umami tastes are mediated via G-protein-coupled receptors, T1Rs and T2Rs, found in type II taste receptor cells [44]. Bitter ligands are detected by T2R of which there are currently over 25 genes encoding the T2Rs [44,45]. Salty and sour taste have been suggested to be modulated by specialised ion channels. Salty taste has been proposed to involve the selective epithelial type sodium channel (ENaC), and putative sour taste receptors include H+ ions permeating type III sour sensing cells resulting in type III sour cells depolarising and reaching action potential [8,44,45], see Roper et al (2017) for a recent comprehensive review on taste receptor mechanisms. 

Umami was widely accepted as a basic taste based on the discovery that the heterodimeric G-protein-coupled receptors, T1R1/T1R3 mediate umami taste detection [17,46,47]. The umami taste heterodimer complex, T1R1/T1R3, shares a common receptor subunit (T1R3) with sweet taste detected by the heterodimeric G-protein-coupled receptors, T1R2/T1R3 [9,44]. T1R1/T1R3 heterodimeric receptor is specific to detecting umami-tasting stimuli (L-amino acids), as it is non-responsive to sweet stimuli but responsive to umami stimuli (MSG and l-glutamate) *in vitro* [17,46]. T1R1/T1R3 was confirmed to respond to umami-tasting stimuli upon the discovery that T1R1, T1R3, and T1R1/T1R3 knockout mice lack, or have attenuated taste responses to umami stimuli (MSG) [47], and human T1R1/T1R3 receptors responded when l-glutamate was applied *in vitro* [46]. Although, studies have found in T1R1 and T1R3 knockout mice that a reduced, but not abolished, taste response to umami stimuli (MSG and MPG) occurs, indicating that other receptors responding to umami stimuli exist [48,49,50,51]. 

When investigating the umami taste synergism occurring from the mixing of IMP/GMP with MSG, T1R3 knockout mice had only moderately reduced taste responses, both neural and behavioural, although the contribution of Na+ was not eliminated in this study, so remaining taste responses in these knockout mice is likely due to the Na+ [48]. Zhao and colleagues showed, in independently generated T1R3 knockout mice, that when the contribution of Na+ was reduced with amiloride, the T1R3 knockout mice lacked responses to IMP with MSG, where responses in control mice remained, highlighting the importance of the T1R3 subunit in umami taste synergism [47]. Similarly, in T1R1 knockout mice the umami synergy when IMP was applied with MSG was abolished [52]. All of this shows that the T1R1/T1R3 umami receptors are important, if not essential, for the synergistic effect of IMP/GMP when applied with MSG, but for MSG in isolation an umami taste response, albeit reduced, remains in the absence of the T1R1/T1R3 umami receptors [53]. This suggests that additional receptors respond to umami taste stimuli, which was supported by studies finding putative umami receptors, metabotropic glutamate receptor 1 and 4 (mGluR1, mGluR4) were activated by concentrations of umami stimuli (MPG) commonly found in food in an *in vitro* assay [51], and mGluR4 knockout mice had reduced neural responses *in vivo* to umami stimuli (MPG) [54]. 

Finally, the discovery of single nucleotide polymorphisms on human TAS1R1, and TAS1R3 receptor genes, and their association with individual variation in umami (MSG, MPG, and MSG+IMP) taste perception phenotypes, provided further evidence for T1R1/T1R3 contributing to umami taste detection in humans [24,55,56].

### 5.2. Neural Responses to Umami Stimuli

When taste receptor cells detect chemicals in the oral cavity a neurotransmitter (ATP) is released onto afferent gustatory fibres, three predominant gustatory afferent nerves transmit information from taste buds to the brain [8]. The 7th cranial nerve, chorda tympani (CT), innervates the anterior two thirds of the tongue, and the 9th cranial nerve, glossopharyngeal (GL), innervates the posterior third, and the 10th cranial nerve, vagus nerve, similarly innervates the posterior of the tongue. The information transmitted for umami taste is then processed in the primary and secondary gustatory cortex [57].

Studies investigating responses of the CT in both wild-type mice (not genetically modified), and T1R3 knockout mice, have shown that there are two predominant fibre groups in the CT [58]. These fibres noted are sucrose best (S) and MPG best, or l-glutamate best (M) fibres, each of these fibres have sub-groups (S1, S2, and M1, M2) [58]. S1 and M1 show synergism between l-glutamate and IMP, whereas S2 and M2 do not display this synergism [58]. In T1R3 knockout mice S1 fibres were lacking, and no synergistic effect between MPG and IMP was observed [58]. Similarly, whole CT responses in T1R3 knockout mice showed the synergism between IMP mixed with MSG is attenuated [48], or eliminated [47], demonstrating the importance of the T1R3 subunit for the synergistic effect between l-glutamate and IMP in the CT nerve. In response to MSG in isolation, T1R3 knockout mice showed reduced CT responses only at the highest MSG concentration [48]. This reduced response did not occur in the GL nerve, indicating that perhaps other receptors mediate umami responses from the GL nerve, for example, the mGluR4 receptor [48]. Supporting this, mGluR4 knockout mice displayed reduced responses to umami stimuli (MPG) in both the CT and GL nerves, these receptors may not be innervated by S1, or S2 fibres [54]. Whether the transduction of umami taste results in a uniquely perceptible experience will be discussed below. 

## 6. Perceptual Independence of Umami Taste 

Perception is input from the senses giving rise to a conscious experience of the particular stimulus [11]. Basic tastes should elicit perceptions independent to other basic tastes, and should not be produced by combination of existing basic tastes, or other sensory systems, such as the somatosensory system (i.e., mouthfeel or mouthfullness) [10,12]. The detection of a compound by taste receptors, and transduction to gustatory processing areas may produce a taste perception, but this taste perception may not always be a perceptually salient experience.

An important point to note regarding perceptual independence of umami is that the compound responsible, l-glutamate, is not used in the glutamic acid form as it is sour, so the sodium salt form is primarily used in psychophysical studies, meaning some potential overlap with salt taste (please see *Umami and salty* section).

Describing the perception arising from tasting umami effective stimuli becomes difficult due to the absence of a clear set of lexicon for describing umami, thus, whether umami is perceptually salient is not clear. Throughout the literature, a multitudinous lexicon has been used to describe umami taste, ranging from meaty, savoury, brothy, mouthfullness, and delicious [35,55,59]. Familiarisation or a learning effect for umami taste perception is not consistent within the literature, for example, a learning effect for umami hypotasters occurred after repeated exposure [60], contrary to this, umami taste sensitivity either increased or decreased depending on participants’ age over repeated measures [61]. Using familiarisation or repeated exposure for improving perceptual salience of umami taste requires further research, as the current literature is inconclusive. The question that remains is whether a basic taste should require familiarisation for a perceptually salient experience to occur? Additionally, the common description of umami flavour as ‘mouthfeel’ [62] implies a tactile component to umami taste, similar to the description used for kokumi taste. Descriptions for umami taste cited within the literature are similar to those used to describe kokumi taste. Kokumi descriptions include deliciousness, rich, continuity, and mouthfullness [63]. Although kokumi is not a basic taste, there are similarities in both effective stimuli (glutamic acid derivatives) and descriptions of perceptual experiences arising from tasting both kokumi and umami stimuli suggests these taste qualities have perceptual similarities. 

Considering l-glutamate is the predominant umami stimuli that is detected by glutamate receptors [17], it may be suitable to predict that foods containing high concentrations of free l-glutamate would ultimately lead to an experience that is perceived, and described, as umami or savoury. Although, it is important to note that taste perception of whole foods is indeed complex, with contribution of many tastants within the one food resulting in the overall taste perception produced. Nevertheless, in foods including seaweed and specific mushrooms, for example shitake, that contain high concentrations of l-glutamate, these are typically described as umami tasting. Contrary to this, there is a number of natural foods containing high concentrations of free l-glutamate that are not described as having an umami taste, for example peas (200 mg/10 g), corn (140 mg/100 g), red grapes (184 mg/100 g), or tomatoes (140 mg/100 g) [14,64,65]. Similar to sweet and sour tastants in the previously mentioned foods, the presence of l-glutamate in these foods is an important compound to produce the overall flavour of these foods, rather than eliciting a clear perceptible umami taste [36,66].

Umami (MSG) has been shown to exhibit partially independent taste perception, as previous studies using multidimensional scaling have found that umami lies perceptually outside of the four basic tastes (sweet, sour, salty, and bitter) (cited in [25]), and that the taste perception of umami is predominately due to the anion (l-glutamate), albeit a small effect of the cation needs to be considered [23]. Perceptual associations between umami and salty taste exist as thresholds for the two tastes were found to correlate in participants classified as umami hypotasters [60]. Perceptual associations may similarly exist for umami and sweet tastes possibly due to the shared taste receptor subunit (T1R3). For example, rodents have reduced discrimination ability between sucrose and MSG when the sodium in MSG is neutralised using a salt taste blocker (amiloride) [49]. Furthermore, in humans, perceptual associations have been found between umami and sweet tastes in umami hyposensitive participants [67]. Therefore, it is pertinent to consider the perceptual relationship between umami taste and basic tastes, specifically salty and sweet tastes. 

### 6.1. Umami and Salty

Glutamate in isolation from the sodium ion is glutamic acid, and has been described as having a sour taste [14]. The sodium salt of l-glutamate, MSG, produces an umami taste, and is the predominant prototypical umami stimulus used in psychophysical testing [60,67,68,69]. MSG potentiating 5’ribonucleotides are similarly tasted in their disodium salt form [13,22] complicating the perceptual independence of umami from salt taste in psychophysical testing [60,67,68,69]. Participants confuse umami with salty taste [68], and food (soup) containing MSG+IMP has been perceived as saltier, but not more savoury, than soups without MSG+IMP [43]. To overcome the sodium component of MSG, MPG is used in some psychophysical studies as a sodium free umami stimuli [55], although potassium also imparts salty, bitter, and metallic tastes, thus for all psychophysical testing l-glutamate requires a cation to produce a perceptible umami taste [14,23].

Through measuring DT and suprathreshold intensity for umami (MSG) and salty (NaCl), Lugaz, Pillias and Faurion (2002) found that 27% of their study population were classified as putative umami hypotasters. This proportion of umami hypotasters is consistent throughout the literature, with 28% of female participants [67], and 21% of participants [55], having a reduced ability to discriminate between 29 mM MSG and 29 mM NaCl. Using a filter paper disk method, 24% of female Japanese subjects had umami taste thresholds above 50 mM MSG, and were considered hypotasters [69]. The remainder of participants were classified as either semi-discriminators [67], or were able to discriminate between NaCl and MSG at the level of significance and were considered umami tasters [55,60]. Although a very low percentage of the population, 3.5% of a French [60], 3.2% of a German, and 4.6% of a Norwegian population [70] had no ability to discriminate between 29 mM NaCl and 29 mM MSG. These participants were unable to taste the l-glutamate in MSG and were considered non-tasters. Whether a similar proportion of subjects would be found to be umami non-tasters and tasters in non-European populations, or populations with high MSG intake, requires further research. 

In individuals with an ability to taste l-glutamate, umami and salty taste perception are independent, conversely, in participants considered umami hypotasters, umami and salty taste perception are associated. Lugaz and colleagues (2002) found a positive correlation (*r* = 0.75) between individual salty (NaCl), and umami taste (MSG) thresholds in hypotasters, indicating that the hypotasters were likely to be perceiving only the sodium cation of the MSG. Along the same line, Pepino and colleagues (2010) found that participants classified as umami hypotasters (referred to as non-discriminators), perceived significantly more saltiness, and significantly less savouriness in MSG at suprathreshold concentrations, than umami tasters. There was no significant difference between umami tasters and hypotasters umami (MSG) DT [67], suggesting different mechanisms may mediate umami DT and suprathreshold taste dimensions supporting Lugaz and colleagues’ (2002) findings that thresholds and intensity perception for umami do not necessarily co-vary. Associations between umami DT and salty DT were not investigated, therefore, it is unknown if an association would have occurred between salty and umami DT in this female population group [67]. 

The difficulty with confirming umami taste perception independent from salt taste perception lies in the sodium cation in MSG. This can be overcome in part by the use of MPG, potassium, however has salty, bitter, and metallic taste characteristics [71], and therefore MPG taste perceptions cannot be solely attributed to l-glutamate. The contribution of the sodium ion in MSG can be reduced with the addition of IMP, nevertheless, IMP is tasted in disodium form and although IMP is added to MSG/MPG at subthreshold NaCl concentrations, the presence of sodium cannot be completely negated. Perceptual associations between umami and salty tastes appear to occur specifically in participants classified as umami hypotasters, but not in umami tasters. That is, for umami hyposensitive or non-tasting participants they are predominately sensing the sodium cation within MSG, resulting in associations between salty and umami taste perception for these participants. 

### 6.2. Umami and Sweet

Umami and sweet taste share a common taste receptor subunit, T1R3, therefore there is potential for perceptual associations to exist [67,69]. Mice are capable of discriminating between MSG and sucrose [49], but when amiloride (a sodium blocker) is applied to neutralise the Na+ in MSG, a significant reduction in discrimination ability occurs [49]. Although significantly reduced, discrimination ability of the mice was still above chance, nevertheless, MSG and sucrose have some perceptual associations in rodents when the perceptual influence of Na+ is eliminated [49]. 

Interestingly, in human studies, umami hypotasters (non-discriminators) (27%, *n* = 16), have both significantly lower umami, and significantly lower sweet taste perception at suprathreshold concentrations, compared to umami tasters (discriminators) [67]. At DT, no association between umami (MSG) and sweet (sucrose) DT was found, again indicating different mechanisms may mediate suprathreshold and DT taste perception [67]. Similarly, umami (MSG) hypotasters (23.8%, *n* = 10) have a significantly lower sweet taste sensitivity at RT than umami tasters (*n* = 32) [69]. This suggests a relationship between umami and sweet taste perception. Contrary to these studies, Chen and colleagues (2009) found no significant difference in sweet taste intensity ratings in umami (MPG) insensitive (*n* = 5), and umami sensitive (*n* = 5) subjects. Mixed results could be attributed to different prototypical umami stimulus used (MPG or MSG), or due to the relatively low number of participants (*n* = 10), compared to previous psychophysical studies [67,69]. Similar to the associations between salty and umami taste, sweet and umami taste perception has been found to be perceptually associated in participants considered umami hypotasters, but not in umami tasters. Although the literature is not consistent, the associations found in some studies could conceivably be due to the shared receptor subunit between sweet and umami tastes, T1R3. 

There is enough evidence to question whether umami is perceptually salient, particularly owing to the lexicon used to describe umami taste perception, and the similarities of these descriptions to kokumi taste. Perceptual independence of umami from salty, and sweet, is also unclear as associations exist between umami and salty, and umami and sweet tastes specifically in umami hypotasters. These associations are found across multiple taste dimensions, including DT, RT, and suprathreshold intensity. 

## 7. Umami and Hedonics

MSG in an aqueous solution does not taste pleasant, however, when added to a complex food such as broth, it enhances palatability. For example, in infants the presence of high concentrations of l-glutamate in a breast milk matrix may increase the milks palatability and acceptability [25], and MSG added to a food matrix (soup) is preferred, but in an aqueous solution MSG is aversive [37]. As previously mentioned, free l-glutamate and IMP/GMP are naturally present in a range of foods. Across many cuisines mixtures of foods containing high concentrations of free l-glutamate are combined with foods containing high concentrations IMP/GMP thereby promoting the umami taste synergism and palatability [1]. For example, in Italian cuisine the combination of parmesan (1200 mg/100 g l-glutamate) and beef/pork mince sauce (70 mg and 200 mg/100 g IMP respectively) or parmesan and tomato (120 mg/100 g l-glutamate). Or in Asian cuisines the combination of fish sauce (ranging from 620–1380 mg/100 g l-glutamate) and meat or fish products (ranging from 70–285 mg/100 g IMP) is frequently seen (see [25] for further examples). This combining of foods for enhanced palatability does not often come independent of sodium and kokumi peptides. Taking the previous example, parmesan contains high concentrations of sodium, and kokumi peptides [72], as does soy sauce [73]. Likewise, in studies where ingredients were omitted to determine key taste active compounds within a food, sodium, and free l-glutamate (along with other taste active amino acids) were common key tastants (reviewed in [74]). 

The combination of added MSG and salt (NaCl) increases the acceptance of some foods, including various soup/stocks [75] and rice dishes [76] at certain ratios (usually between 0.1% and 0.8% by weight [66]) depending on the foodstuff and culture. For example, in European populations this may be higher (between 0.6 to 1.2%), possibly owing to the reduced familiarity of umami taste in Western populations [77]. The addition of MSG to improve palatability has been successfully used to reduce the sodium concentration in food without implicating the sensory properties of the foods [75,76], thus, displaying that in certain foods l-glutamate, IMP, sodium and kokumi effective peptides all contribute to the development of flavour and palatability in commonly consumed foods globally. 

## 8. Relationship between Receptor, Perception, and Behavioural Responses of Umami and Sweet Taste

Perceptual associations between sweet and umami taste exist and may be due to behavioural factors including MSG and sucrose consumption [69,78], potentially owing to expression or sensitivity of the shared common receptor subunit T1R3 [17]. *In vitro* when MSG and sucrose are co-applied to sweet taste receptor cells, the response of the sweet taste receptor cells to sucrose is weakened [79]. Response from sweet taste receptor cells is also significantly weakened when glutamyl dipeptides are co-applied with sucrose [79]. When the umami tasting compounds are applied with lactisole, which inhibits activation of T1R3, a more severe reduction in the response from sweet taste receptors occurs. If umami tasting compounds and lactisole interacted with the same transmembrane domain of the T1R3, a synergistic reduction would not be expected, as the two stimuli would be competing for the same transmembrane domain. This suggests an interaction between umami peptides and MSG with sweet taste receptors, preventing sweet substances binding to an alternative domain, potentially T1R2 extracellular domain rather than the T1R3 domain [79]. 

Interestingly, in a human intervention study, prolonged consumption of MSG significantly reduced female participants’ umami suprathreshold intensity perception, and similarly reduced (trending towards significant, *p* = 0.06) sweet taste suprathreshold intensity perception [78]. Similarly, Kubota and colleagues (2018) found that umami hypotasters also had a decreased sweet taste sensitivity, and consumed more sugar than umami tasters, although causation cannot be inferred between umami perception, sweet taste perception and sugar intake. It was not investigated whether umami hypotasters simply had a lower taste sensitivity overall compared to umami tasters, although this is unlikely as no significant differences in bitter taste sensitivity between umami tasters, and non-tasters was found [69]. 

Increased consumption has been linked with decreased receptor expression for other basic tastes, for example, increased consumption of fat was associated with decreased fat taste perception and decreased expression of fat taste receptor CD36 [18,80]. It would be interesting to know if increased intake of umami and sweet tastes decreases both taste perception of sweet and umami tastes and expression of the shared receptor subunit, T1R3. Alternatively, it is possible that increased intake of l-glutamate may decrease expression of T1R1, mGluR1, or mGluR4 taste receptors, although this does not account for the reduction in sweet taste perception found in previous psychophysical studies [69,78]. Considering umami stimuli may interact with the T1R2 extracellular domain [79], it would be interesting to investigate the influence of oral exposure to umami on T1R2 receptor expression. Although further research into receptor expression is required, dietary intake of both sweet and umami stimuli appear to influence both umami and sweet taste perception in a similar direction, showing an interesting association between receptor, perception, and intake for umami and sweet tastes. 

## 9. Behavioural and Physiological Responses to Umami Effective Stimuli

A key aspect of taste and taste receptor activation is the physiological responses initiated from oral taste receptor activation, and the influence on behavioural responses in human studies, for example increasing satiation and satiety [41,81]. The commencement of digestion is initiated through the secretion of saliva, the presence of MSG in the oral cavity stimulates a strong response of salivary release through a vagal efferent activation, assisting in initiating this digestion [40,82]. l-glutamate is not only detected in the oral cavity, but also in the gastrointestinal tract where T1R1/T1R3 are found [40,42,83]. T1R1/T1R3 heterodimer has been suggested to affect nutrient absorption through regulation of a peptide transporter through the activation by l-glutamate (reviewed in [42]). Daly and colleagues (2013) found in rodents’ gastrointestinal tract T1R1/T1R3 are expressed and activation by l-glutamate results in CCK secretion *in vitro*, which is enhanced by IMP. CCK is involved in digestive processes, including slowing gastric emptying, and has also been suggested to inhibit food intake, thus has a satiety-like action through activating vagal afferent fibres that innervate the stomach and upper intestine (reviewed in [84]). 

This enhanced satiety-like action from glutamate consumption has been demonstrated in human behavioural studies where the return of hunger after eating is slowed down after participants consume soup containing MSG, compared to soup without MSG [85], and soup containing protein and MSG compared to other treatments [86]. Similarly, in infants, consumption is decreased and satiation and satiety is increased when infants are fed formula supplemented with MSG, compared with standard cow’s milk formula with the same concentration of protein [87]. Masic et al (2014) postulated that umami flavour may play a role in the satiating effects of protein, through sensory-nutrient interactions. Conversely, pre-load soups all containing MSG + IMP in conjunction with either low-energy, high-energy carbohydrate, or high-energy protein, all reduced consumption at a subsequent test meal compared to the same pre-load soups without added MSG + IMP [43]. The presence of MSG + IMP alone reduced consumption, irrespective of the protein content of the preload soup [43]. It is plausible that the presence of MSG + IMP enhanced the post-ingestive release of CCK in the gastrointestinal tract, influencing gastric emptying for all soup pre-load conditions, enhancing satiety, and reducing subsequent intake, although this requires further research. The effect of MSG on satiety is not consistent in the literature, as pre-load soups containing MSG improved energy compensation at a subsequent test meal but did not reduce hunger ratings or total energy intake compared to pre-load soups without MSG [88]. This indicates the importance of IMP in conjunction with MSG, for satiety-like responses and potentially CCK secretion, and considering MSG and IMP are often consumed together in animal protein and other common food combinations, this provides evidence for the importance of umami taste detection and perception in physiological processes and behavioural outcomes. 

Interestingly, when a combination of tastants (sweet, bitter, and umami) were infused directly into the duodenum, increased satiety and decreased hunger responses were observed, as was a reduction in consumption of an *ad libitum* meal [89]. When umami was infused in isolation this reduction in hunger and increase in satiety was still observed, but not when sweet or bitter were infused alone, demonstrating the results from the combination of tastants were predominately driven by umami, with the exception of reducing energy intake at an *ad libitum* meal. Interestingly, the infusion of all individual tastants and combination of tastants did not influence the secretion of gastrointestinal peptides in comparison to the placebo infusion. It would be interesting to investigate the interaction of umami stimuli with taste receptors in the oral cavity without subsequent consumption, and whether individual variation in umami taste sensitivity is associated with the previously discussed physiological (satiety hormone release) and behavioural responses (satiety, satiation and intake). Although further research is required, the consumption of MSG and IMP and the discovery of glutamate receptors in the gastrointestinal tract provides evidence for the role of umami effective stimuli detection stimulating physiological responses which may translate into behavioural responses in human studies. 

## 10. Summary—Is Umami A Basic Taste?

**Figure 1:**
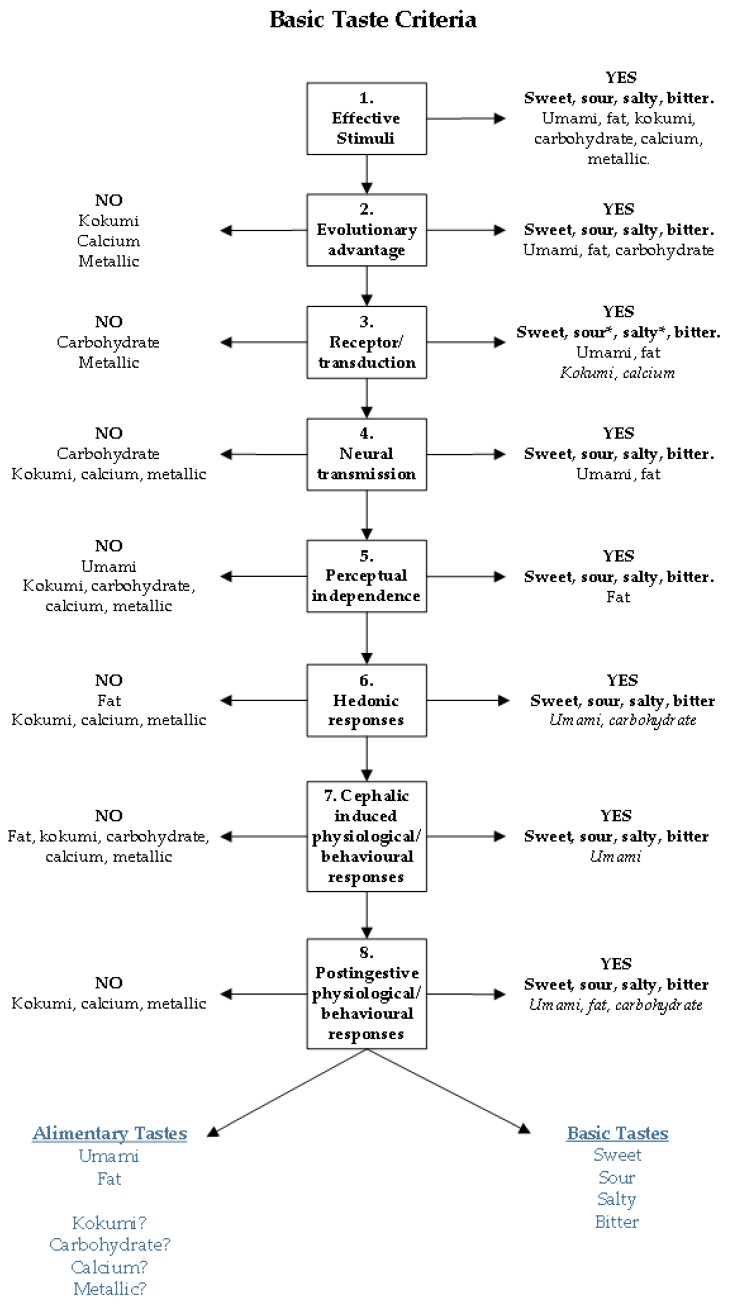
Criteria for tastes to fulfil to be classified as either basic tastes, or within a new taste subgroup. At the first criteria that a taste does not fulfil it is placed on the left-hand side of the model in the ‘NO’ section, those that fulfil the criteria remain on the right-hand side in the ‘YES’ criteria. * ENaC knockout mice have eliminated taste and neural responses to NaCl providing evidence for ENaC as the salt taste receptor [90], human studies have not yet confirmed the ENaC channel for salt taste detection. For the receptor criterion the ENaC receptor for salt taste, albeit in mice, has supporting evidence. * Type III sour sensing cells have been shown depolarise and reach action potential due to influx of H+ ions, providing evidence for sour taste detection, the specific proton channel responsible for this remains to be confirmed [45].

For the past 3000 years four tastes (sweet, sour, salty, and bitter) have been included in all lists of basic tastes, predominantly based on perception. While these lists changed significantly in other attributes listed, often dependent on current thinking at that time, sweet, sour, salty, and bitter have been consistent. The recent advancements of technology and knowledge has led to the discovery of taste receptors and ligands, extending this basic taste list to include umami and fat as basic tastes. Thus, the basic taste list has grown and has the potential to include a plethora of other tastes including kokumi, carbohydrate, calcium and metallic tastes. With the potential of an ever-growing list of basic tastes in the current day it is pertinent to evaluate the current evidence and the ‘moment in time’ approach to naming basic tastes. It seems reasonable for new basic tastes, including umami, to consider if they belong in the same category as sweet, sour, salty, and bitter. Below is a summary of the evidence of umami as a basic taste, including an overview of basic and new tastes, against the proposed taste criteria, see Figure 1.

1. *Having an evolutionary or adaptive advantage*: Yes. Umami taste appears to have a biphasic effect due to its involvement in appetite stimulation and then digestion regulation. This occurs through both increasing satiety [43], and the presence of glutamate receptors in the gastrointestinal tract stimulating the release of digestive hormones [40,41,42], providing evidence for umami taste perception existing for evolutionary purposes.
2. *A distinct class of effective stimuli must exist:* Yes. Unique umami effective stimuli found in food includes free l-glutamate, and 5’ribonucleotides, and the prototypical umami taste stimuli are the salts of glutamic acid, MSG or MPG, and disodium salts of IMP and GMP [14,23]. There are a number of foods high in free l-glutamate that would not commonly be described as umami, raising the question of whether high concentrations of naturally occurring l-glutamate elicits an umami like taste in all foods [14,64,65]. Although, common food processing such as curing, and ageing, can increase free l-glutamate and IMP in certain foods, enhancing the umami taste through the glutamate and IMP synergism [26]. Finally, there is similarity between kokumi and umami stimuli, predominately due to the involvement of glutamic acid derivatives [30].
3. *Transduction mechanisms that can convert the chemical code of the stimulus into an electrical signal is required, including receptors:* Yes. Glutamate taste receptors have been identified (T1R1/T1R3, mGluR1, and mGluR4), and these respond to umami stimuli [17,46,47]. This glutamate taste receptor heterodimer (T1R1/T1R3), shares a receptor subunit with the sweet taste receptor (T1R2/T1R3) which has been hypothesised to relate to the perceptual associations that has been found between sweet and umami taste [69,78].
4. *Neurotransmission of this electrical signal to processing regions of the brain must occur:* Yes. Neurotransmission of signals transduced from glutamate receptors occurs, interestingly evidence suggests that different stimuli (MSG, MPG, MSG+IMP) are transduced by different gustatory afferent nerves (CT, GL) for umami taste.
5. *Perceptual experience arising from this processing must be independent from other taste qualities:* No. Studies using multidimensional scaling have found that umami lies perceptually outside of the four basic tastes (sweet, sour, salty, and bitter) (cited in [25]), and individual variation in taste perception across multiple taste dimensions has been established. Nevertheless, prototypical umami stimuli (l-glutamate or IMP/GMP) require cations to produce an umami taste, regardless of whether this cation is sodium or potassium, the additional taste that is imparted is difficult to negate in psychophysical testing. Studies have found perceptual associations with umami and salty taste, specifically in participants considered umami hypotasters at DT [60], and increased saltiness perception of MSG at suprathreshold concentrations [67]. For umami and sweet taste, associations have similarly been found at DT [67] and RT [69], possibly owing to the shared receptor subunit T1R3. Considering current research finds perceptual associations between umami, other basic tastes (salty and sweet) and putative tastes (kokumi), it is relevant to question umami’s classification as a basic taste. Perhaps umami taste fits into a taste classification with other basics (fat) or putative tastes including carbohydrate, kokumi, metallic, and calcium tastes that do elicit a taste perception when presented at high enough concentrations in the oral cavity but this is not necessarily a unique or perceptually salient taste experience.
6. *Hedonic response from tasting umami stimuli:* Yes. Although in aqueous solution MSG is not pleasant in taste, when mixed to certain foods it enhances palatability. The combination of l-glutamate, IMP, sodium, and often kokumi peptides is important in enhancing palatability of certain foods and is found across many cuisines globally.
7. *Physiological effects must occur following activation of taste bud cells:* Yes. Free l-glutamate is not only detected in the oral cavity, but also in the gastrointestinal tract where glutamate taste receptors (T1R1/T1R3) are present [40,42,83]. Glutamate taste receptor heterodimers have been suggested to affect nutrient absorption through regulation of a peptide transporter and glucose transporter through the activation of T1R1/T1R3 by l-glutamate (reviewed in [42]), which also results in CCK secretion *in vitro* [41]. Although the findings in the literature is mixed, behavioural studies have shown that consumption of MSG and particularly MSG+IMP influences satiety, satiation, and food intake, possibly owing to the secretion of digestive peptides upon stimulation of glutamate receptors in the gastrointestinal tract.

## 11. A New Class for New Tastes: Alimentary Taste

Current advances in knowledge and technology has led to the discovery of taste receptors, which has broadened the stimuli that could potentially be considered basic tastes, including kokumi, calcium, and likely many more to be discovered, for example, receptors responding to carbohydrate and metallic taste ligands. So, this ‘moment in time’ list of basic tastes has begun to expand with the addition of umami and fat, and others on the horizon such as carbohydrate and kokumi. Should kokumi, fat, or even umami be classified in the same category as sweet, sour, salty, and bitter, all of which have lingered throughout history? Is it enough to have identified receptors on taste cells for new tastes to be considered a basic taste, if the activation of these receptors does not result in a perceptually independent (umami) or perceptually salient (umami and fat) experience? An example of the identification of receptors with an absence of perceptual salience is fat taste, conceivably umami may fall into a similar category. Due to their unquestionable perceptual salience, sweet, sour, salty, and bitter have importance for immediate decision making; do we ingest or reject, that is, these tastes are critical during pre-ingestive taste detection. Many of the new and putative tastes may have far greater importance on post-ingestive consequences of nutrients that are detected not only in the oral cavity, but throughout the alimentary canal. Perhaps it is important, particularly in the context of applied taste research, that we consider umami and fat in a new subgroup of tastes. We propose a new structure of taste classification, with the four traditional tastes remaining as basic tastes due to their critical function during pre-ingestive taste detection, and new tastes becoming ‘alimentary’ tastes, including umami and fat, which have greater importance for post-ingestive functioning. 

## References

[1] Kurihara K. (2009). Glutamate: From discovery as a food flavor to role as a basic taste (umami). Am. J. Clin. Nutr..

[2] Newman L., Keast R. (2013). The Test–Retest Reliability of Fatty Acid Taste Thresholds. Chemosens. Percept..

[3] Dunkel A., Köster J., Hofmann T. (2007). Molecular and Sensory Characterization of γ-Glutamyl Peptides as Key Contributors to the Kokumi Taste of Edible Beans (*Phaseolus vulgaris* L.). J. Agric. Food Chem..

[4] Ueda Y., Yonemitsu M., Tsubuku T., Sakaguchi M., Miyajima R. (1997). Flavor Characteristics of Glutathione in Raw and Cooked Foodstuffs. Biosci. Biotechnol. Biochem..

[5] Low J.Y.Q., Lacy K.E., McBride R.L., Keast R.S.J. (2017). Evidence supporting oral sensitivity to complex carbohydrates independent of sweet taste sensitivity in humans. PLOS ONE.

[6] Tordoff M.G. (2001). Calcium: Taste, intake, and appetite. Physiol. Rev..

[7] Lawless H.T., Stevens D.A., Chapman K.W., Kurtz A. (2005). Metallic taste from electrical and chemical stimulation. Chem. Senses.

[8] Chaudhari N., Roper S.D. (2010). The cell biology of taste. J. Cell Biol..

[9] Chandrashekar J., Hoon M.A., Ryba N.J.P., Zuker C.S. (2006). The receptors and cells for mammalian taste. Nature.

[10] Keast R.S., Costanzo A. (2015). Is fat the sixth taste primary? Evidence and implications. Flavour.

[11] Breslin P.A.S. (2013). An Evolutionary Perspective on Food Review and Human Taste. Curr. Biol..

[12] Mattes R.D. (2011). Accumulating Evidence Supports a Taste Component for Free Fatty Acids in Humans. Physiol. Behav..

[13] Kurihara K., Kashiwayanagi M. (2000). Physiological Studies on Umami Taste. J. Nutr..

[14] Kurihara K. (2015). Umami the Fifth Basic Taste: History of Studies on Receptor Mechanisms and Role as a Food Flavor. BioMed Res. Int..

[15] Bartoshuk L.M., Carterette E.C., Friedman M.P. (1978). History of Taste Research.

[16] McBurney D.H., Gent J.F. (1979). On the nature of taste qualities. Psychol. Bull..

[17] Nelson G., Chandrashekar J., Hoon M.A., Feng L., Zhao G., Ryba N.J.P., Zuker C.S. (2002). An amino-acid taste receptor. Nature.

[18] Liu D., Archer N., Duesing K., Hannan G., Keast R. (2016). Mechanism of fat taste perception: Association with diet and obesity. Prog. Lipid Res..

[19] Ohsu T., Amino Y., Nagasaki H., Yamanaka T., Takeshita S., Hatanaka T., Maruyama Y., Miyamura N., Eto Y. (2010). Involvement of the Calcium-sensing Receptor in Human Taste Perception. J. Biol. Chem..

[20] Low J.Y.Q., Lacy K.E., McBride R.L., Keast R.S.J. (2017). Carbohydrate Taste Sensitivity Is Associated with Starch Intake and Waist Circumference in Adults. J. Nutr..

[21] Ikeda K. (2002). New Seasonings. Chem. Senses.

[22] Yamaguchi S. (1967). The Synergistic Taste Effect of Monosodium Glutamate and Disodium 5′-Inosinate. J. Food Sci..

[23] Yamaguchi S. (1991). Basic properties of umami and effects on humans. Physiol. Behav..

[24] Shigemura N., Shirosaki S., Sanematsu K., Yoshida R., Ninomiya Y. (2009). Genetic and molecular basis of individual differences in human umami taste perception. PLoS ONE.

[25] Yamaguchi S., Ninomiya K. (2000). Umami and Food Palatability. J. Nutr..

[26] Ninomiya K. (1998). Natural occurrence. Food Rev. Int..

[27] Agostoni C., Carratu B., Boniglia C., Lammardo A.M., Riva E., Sanzini E. (2000). Free glutamine and glutamic acid increase in human milk through a three-month lactation period. J. Pediatr. Gastroenterol. Nutr..

[28] Toelstede S., Hofmann T. (2009). Kokumi-Active Glutamyl Peptides in Cheeses and Their Biogeneration by Penicillium roquefortii. J. Agric. Food Chem..

[29] Kuroda M., Kato Y., Yamazaki J., Kai Y., Mizukoshi T., Miyano H., Eto Y. (2012). Determination and Quantification of γ-Glutamyl-valyl-glycine in Commercial Fish Sauces. J. Agric. Food Chem..

[30] Paolella S., Prandi B., Falavigna C., Buhler S., Dossena A., Sforza S., Galaverna G. (2018). Occurrence of non-proteolytic amino acyl derivatives in dry-cured ham. Food Res. Int..

[31] Steiner J.E., Glaser D., Hawilo M.E., Berridge K.C. (2001). Comparative expression of hedonic impact: Affective reactions to taste by human infants and other primates. Neurosci. Biobehav. Rev..

[32] Peng Y., Gillis-Smith S., Jin H., Tränkner D., Ryba N.J., Zuker C.S. (2015). Sweet and bitter taste in the brain of awake behaving animals. Nature.

[33] Beauchamp G.K. (2016). Why do we like sweet taste: A bitter tale?. Physiol. Behav..

[34] Newman L., Haryono R., Keast R. (2013). Functionality of Fatty Acid Chemoreception: A Potential Factor in the Development of Obesity?. Nutrients.

[35] Beauchamp G.K. (2009). Sensory and receptor responses to umami: An overview of pioneering work. Am. J. Clin. Nutr..

[36] Ninomiya K. (2002). Umami: A universal taste. Food Rev. Int..

[37] Vazquez M., Pearson P.B., Beauchamp G.K. (1982). Flavor preferences in malnourished Mexican infants. Physiol. Behav..

[38] Beauchamp G.K., Pearson P. (1991). Human development and umami taste. Physiol. Behav..

[39] Kondoh T., Mallick H.N., Torii K. (2009). Activation of the gut-brain axis by dietary glutamate and physiologic significance in energy homeostasis. Am. J. Clin. Nutr..

[40] San Gabriel A., Uneyama H. (2013). Amino acid sensing in the gastrointestinal tract. Amino Acids.

[41] Daly K., Al-Rammahi M., Moran A., Marcello M., Ninomiya Y., Shirazi-Beechey S.P. (2013). Sensing of amino acids by the gut-expressed taste receptor T1R1–T1R3 stimulates CCK secretion. Am. J. Physiol..

[42] Wauson E.M., Lorente-Rodríguez A., Cobb M.H. (2013). Minireview: Nutrient Sensing by G Protein-Coupled Receptors. Mol. Endocrinol..

[43] Masic U., Yeomans M.R. (2014). Umami flavor enhances appetite but also increases satiety. Am. J. Clin. Nutr..

[44] Bachmanov A.A., Beauchamp G.K. (2007). Taste Receptor Genes. Annu. Rev. Nutr..

[45] Roper S.D., Chaudhari N. (2017). Taste buds: Cells, signals and synapses. Nat. Rev. Neurosci..

[46] Li X., Staszewski L., Xu H., Durick K., Zoller M., Adler E. (2002). Human receptors for sweet and umami taste. Proc. Natl. Acad. Sci. USA.

[47] Zhao G.Q., Zhang Y., Hoon M.A., Chandrashekar J., Erlenbach I., Ryba N.J.P., Zuker C.S. (2003). The receptors for mammalian sweet and umami taste. Cell.

[48] Damak S., Rong M., Yasumatsu K., Kokrashvili Z., Varadarajan V., Zou S., Jiang P., Ninomiya Y., Margolskee R.F. (2003). Detection of sweet and umami taste in the absence of taste receptor T1r3. Science.

[49] Delay E.R., Hernandez N.P., Bromley K., Margolskee R.F. (2006). Sucrose and monosodium glutamate taste thresholds and discrimination ability of T1R3 knockout mice. Chem. Senses.

[50] Maruyama Y., Pereira E., Margolskee R.F., Chaudhari N., Roper S.D. (2006). Umami Responses in Mouse Taste Cells Indicate More than One Receptor. J. Neurosci..

[51] Pal Choudhuri S., Delay R.J., Delay E.R. (2016). Metabotropic glutamate receptors are involved in the detection of IMP and l-amino acids by mouse taste sensory cells. Neuroscience.

[52] Kusuhara Y., Yoshida R., Ohkuri T., Yasumatsu K., Voigt A., Hübner S., Maeda K., Boehm U., Meyerhof W., Ninomiya Y. (2013). Taste responses in mice lacking taste receptor subunit T1R1. J. Physiol..

[53] Blonde G.D., Travers S.P., Spector A.C. (2018). Taste sensitivity to a mixture of monosodium glutamate and inosine 5′-monophosphate by mice lacking both subunits of the T1R1+T1R3 amino acid receptor. Am. J. Physiol.-Regul. Integr. Comp. Physiol..

[54] Yasumatsu K., Manabe T., Yoshida R., Iwatsuki K., Uneyama H., Takahashi I., Ninomiya Y. (2014). Involvement of multiple taste receptors in umami taste: Analysis of gustatory nerve responses in metabotropic glutamate receptor 4 knockout mice. J. Physiol..

[55] Chen Q.-Y., Alarcon S., Tharp A., Ahmed O.M., Estrella N.L., Greene T.A., Rucker J., Breslin P.A.S. (2009). Perceptual variation in umami taste and polymorphisms in TAS1R taste receptor genes. Am. J. Clin. Nutr..

[56] Raliou M., Grauso M., Hoffmann B., Schlegel–Le-Poupon C., Nespoulous C., Débat H., Belloir C., Wiencis A., Sigoillot M., Preet Bano S. (2011). Human Genetic Polymorphisms in T1R1 and T1R3 Taste Receptor Subunits Affect Their Function. Chem. Senses.

[57] De Araujo I.E.T., Kringelbach M.L., Rolls E.T., Hobden P. (2003). Representation of umami taste in the human brain. J. Neurophysiol..

[58] Yasumatsu K., Ogiwara Y., Takai S., Yoshida R., Iwatsuki K., Torii K., Margolskee R.F., Ninomiya Y. (2012). Umami taste in mice uses multiple receptors and transduction pathways. J. Physiol..

[59] Chaudhari N., Pereira E., Roper S.D. (2009). Taste receptors for umami: The case for multiple receptors. Am. J. Clin. Nutr..

[60] Lugaz O., Pillias A.M., Faurion A. (2002). A new specific ageusia: Some humans cannot taste L-glutamate. Chem. Senses.

[61] Mojet J., Christ-Hazelhof E., Heidema J. (2001). Taste perception with age: Generic or specific losses in threshold sensitivity to the five basic tastes?. Chem. Senses.

[62] Prescott J. (2004). Effects of added glutamate on liking for novel food flavors. Appetite.

[63] Yamamoto T., Watanabe U., Fujimoto M., Sako N. (2009). Taste preference and nerve response to 5′-inosine monophosphate are enhanced by glutathione in mice. Chem. Senses.

[64] Löliger J.R. (2000). Function and Importance of Glutamate for Savory Foods. J. Nutr..

[65] Giacometti T. (1979). Free and Bound Glutamate in Natural Products. Glutamic Acid.

[66] Jinap S., Hajeb P. (2010). Glutamate. Its applications in food and contribution to health. Appetite.

[67] Pepino M.Y., Finkbeiner S., Beauchamp G.K., Mennella J.A. (2010). Obese Women Have Lower Monosodium Glutamate Taste Sensitivity and Prefer Higher Concentrations Than Do Normal-weight Women. Obesity.

[68] Overberg J., Hummel T., Krude H., Wiegand S. (2012). Differences in taste sensitivity between obese and non-obese children and adolescents. Arch. Dis. Child..

[69] Kubota M., Toda C., Nagai-Moriyama A. (2018). Relationship between umami taste acuity with sweet or bitter taste acuity and food selection in Japanese women university students. Asia Pac. J. Clin. Nutr..

[70] Singh P.B., Schuster B., Seo H.S. (2010). Variation in umami taste perception in the German and Norwegian population. Eur. J. Clin. Nutr..

[71] Sinopoli D.A., Lawless H.T. (2012). Taste Properties of Potassium Chloride Alone and in Mixtures with Sodium Chloride Using a Check-All-That-Apply Method. J. Food Sci..

[72] Sforza S., Cavatorta V., Galaverna G., Dossena A., Marchelli R. (2009). Accumulation of non-proteolytic aminoacyl derivatives in Parmigiano-Reggiano cheese during ripening. Int. Dairy J..

[73] Kuroda M., Kato Y., Yamazaki J., Kai Y., Mizukoshi T., Miyano H., Eto Y. (2013). Determination and quantification of the kokumi peptide, gamma-glutamyl-valyl-glycine, in commercial soy sauces. Food Chem..

[74] Fuke S., Konosu S. (1991). Taste-active components in some foods: A review of Japanese research. Physiol. Behav..

[75] Jinap S., Hajeb P., Karim R., Norliana S., Yibadatihan S., Abdul-Kadir R. (2016). Reduction of sodium content in spicy soups using monosodium glutamate. Food Nutr. Res..

[76] Leong J., Kasamatsu C., Ong E., Hoi J.T., Loong M.N. (2016). A study on sensory properties of sodium reduction and replacement in Asian food using difference-from—Control test. Food Sci. Nutr..

[77] Bellisle F. (2008). Experimental studies of food choices and palatability responses in European subjects exposed to the Umami taste. Asia Pac. J. Clin. Nutr..

[78] Noel C.A., Finlayson G., Dando R. (2018). Prolonged Exposure to Monosodium Glutamate in Healthy Young Adults Decreases Perceived Umami Taste and Diminishes Appetite for Savory Foods. J. Nutr..

[79] Shim J., Son H.J., Kim Y., Kim K.H., Kim J.T., Moon H., Kim M.J., Misaka T., Rhyu M.R. (2015). Modulation of sweet taste by umami compounds via sweet taste receptor. PLoS ONE.

[80] Costanzo A., Nowson C., Orellana L., Bolhuis D., Duesing K., Keast R. (2018). Effect of dietary fat intake and genetics on fat taste sensitivity: A co-twin randomized controlled trial. Am. J. Clin. Nutr..

[81] Kitamura A., Tsurugizawa T., Torii K. (2011). Biological Significance of Glutamate Signaling during Digestion of Food through the Gut-Brain Axis. Digestion.

[82] Hodson N.A., Linden R.W.A. (2006). The effect of monosodium glutamate on parotid salivary flow in comparison to the response to representatives of the other four basic tastes. Physiol. Behav..

[83] Norton M., Murphy K.G. (2017). Targeting gastrointestinal nutrient sensing mechanisms to treat obesity. Curr. Opin. Pharmacol..

[84] Moran T.H. (2009). Gut peptides in the control of food intake. Int. J. Obes..

[85] Masic U., Yeomans M.R. (2013). Does monosodium glutamate interact with macronutrient composition to influence subsequent appetite?. Physiol. Behav..

[86] Anderson G.H., Fabek H., Akilen R., Chatterjee D., Kubant R. (2018). Acute effects of monosodium glutamate addition to whey protein on appetite, food intake, blood glucose, insulin and gut hormones in healthy young men. Appetite.

[87] Ventura A.K., Beauchamp G.K., Mennella J.A. (2012). Infant regulation of intake: The effect of free glutamate content in infant formulas. Am. J. Clin. Nutr..

[88] Masic U., Yeomans M.R. (2014). Monosodium glutamate delivered in a protein-rich soup improves subsequent energy compensation. J. Nutr. Sci..

[89] van Avesaat M., Troost F.J., Ripken D., Peters J., Hendriks H.F.J., Masclee A.A.M. (2015). Intraduodenal infusion of a combination of tastants decreases food intake in humans. Am. J. Clin. Nutr..

[90] Chandrashekar J., Kuhn C., Oka Y., Yarmolinsky D.A., Hummler E., Ryba N.J., Zuker C.S. (2010). The cells and peripheral representation of sodium taste in mice. Nature.

